# Fabry Cardiomyopathy: Current Practice and Future Directions

**DOI:** 10.3390/cells10061532

**Published:** 2021-06-17

**Authors:** Jeffrey Yim, Olivia Yau, Darwin F. Yeung, Teresa S. M. Tsang

**Affiliations:** 1 Department of Medicine, University of British Columbia, Vancouver, BC V6H 0A5, Canada; jeffyim@alumni.ubc.ca; 2 Faculty of Medicine, University of British Columbia, Vancouver, BC V6H 0A5, Canada; oyau@student.ubc.ca; 3 Vancouver General Hospital and University of British Columbia Echocardiography Laboratory, Division of Cardiology, University of British Columbia, Vancouver, BC V6H 0A5, Canada

**Keywords:** Fabry cardiomyopathy, Fabry disease, lysosomal storage disorder

## Abstract

Fabry disease (FD) is an X-linked lysosomal storage disorder caused by mutations in the galactosidase A (GLA) gene that result in deficient galactosidase A enzyme and subsequent accumulation of glycosphingolipids throughout the body. The result is a multi-system disorder characterized by cutaneous, corneal, cardiac, renal, and neurological manifestations. Increased left ventricular wall thickness represents the predominant cardiac manifestation of FD. As the disease progresses, patients may develop arrhythmias, advanced conduction abnormalities, and heart failure. Cardiac biomarkers, point-of-care dried blood spot testing, and advanced imaging modalities including echocardiography with strain imaging and magnetic resonance imaging (MRI) with T1 mapping now allow us to detect Fabry cardiomyopathy much more effectively than in the past. While enzyme replacement therapy (ERT) has been the mainstay of treatment, several promising therapies are now in development, making early diagnosis of FD even more crucial. Ongoing initiatives involving artificial intelligence (AI)-empowered interpretation of echocardiographic images, point-of-care dried blood spot testing in the echocardiography laboratory, and widespread dissemination of point-of-care ultrasound devices to community practices to promote screening may lead to more timely diagnosis of FD. Fabry disease should no longer be considered a rare, untreatable disease, but one that can be effectively identified and treated at an early stage before the development of irreversible end-organ damage.

## 1. Introduction

Fabry disease is an X-linked lysosomal storage disorder caused by mutations in the α-galactosidase A (*GLA*) gene that result in deficient α-galactosidase A (α-Gal A) enzyme and the accumulation of globotriaosylceramide (Gb_3_) and associated glycosphingolipids throughout the body [1]. The accumulation of Gb_3_ in lysosomes leads to metabolic dysfunction and subsequent cellular death in various organs, leading to a multisystemic clinical presentation that includes cutaneous, corneal, renal, neurological, and cardiac manifestations.

Fabry disease has long been considered a rare disease with limited diagnostic and treatment options. However, the condition is likely underdiagnosed, with newborn screening programs around the world showing much higher prevalence of *GLA* mutation than previously described [2,3]. Furthermore, we now have more effective tools to diagnose FD in a more timely fashion [4,5,6,7]. The development of novel effective therapies has made the early diagnosis of FD and prompt institution of therapy even more important.

The purpose of this review is to provide an overview of the cardiac manifestations of Fabry disease, methods of screening and diagnosis, currently available and investigational treatments, ongoing challenges in management, and future directions to improve the care of patients with this condition.

## 2. Clinical Presentation of Fabry Disease

The typical presentation of Type I ‘classical FD’ is a male patient of the first and second decades of life who presents with acroparesthesia (burning pain in the extremities), gastrointestinal symptoms (including nausea, diarrhea or constipation, and abdominal pain), and angiokeratoma corporis (distinct cutaneous abnormality characterized by vascular papules distributed in the inguinal, hip, and periumbilical areas) [8]. Patients subsequently develop more severe cardiac, renal, and neurologic complications in the third and fourth decades of life [8]. Classical FD has a typical disease onset of childhood or early adolescence and is described in hemizygous male FD patients or heterozygous female FD patients with skewed X-chromosome inactivation of the normal *GLA* allele [9]. Classical FD patients are characterized by a nearly absent level of α-Gal A activity. In contrast to other lysosomal storage diseases, a large number of patients with FD have the late-onset Type II ‘non-classical FD’ phenotype, remaining asymptomatic during very first few decades of life due to residual α-Gal A activity. Recently, sub-classifications of FD including “cardiac variants” with isolated cardiac findings have been identified [10,11].

## 3. Fabry Cardiomyopathy

Cardiac manifestations of FD include increased left ventricular (LV) wall thickness, conduction abnormalities, arrhythmias, valvular disease, and aortic dilatation, which result from glycolipid deposition and subsequent fibrosis of contractile cardiomyocytes, conductive cardiomyocytes, valvular interstitial cells, and smooth muscle cells of the cardiovascular system (Table 1). Eventually, complications such as hypertension, myocardial infarction, and cardiac death may occur, with heart failure being the most common first cardiovascular event in FD [12]. Compared to other organs, the heart appears to be the most susceptible to low levels of α-Gal A. The FD-related cardiovascular injury is thought to be due to a combination of Gb_3_ accumulation, the accumulation of trophic factors, and microcirculatory ischemia, which contribute to inflammation and ultimately result in myocardial fibrosis [13]. Patients with FD-related cardiac involvement tend to be asymptomatic from a cardiac perspective during the first four decades of life, then present with non-specific cardiac symptoms such as angina, dyspnea, palpitations, or syncope. Since there is no pathognomonic cardiac manifestation of FD, the non-specific findings often make FD-related cardiac involvement difficult to diagnose.

The hallmark feature of FD cardiomyopathy is increased LV wall thickness [6,9]. Increased right ventricular wall thickness and impaired right ventricular function have also been reported [16]. Increased LV wall thickness is rarely present in children with FD, tends to be more severe in male FD patients, and is usually not evident until the third or fourth decade in classical FD patients [4,17]. However, the finding of increased LV wall thickness is not specific for Fabry cardiomyopathy, and it is important for clinicians to consider the differential diagnoses of other causes of increased LV wall thickness (Table 2).

Electrophysiologic abnormalities represent other common cardiac manifestations of FD [9]. Advanced conduction disease is thought to be caused by glycolipid accumulation in cardiomyocytes of the atrioventricular (AV) node, bundle of His, and the left and right bundle branches [18]. In contrast, accelerated AV conduction is common in younger FD patients and is reflected as shortened PR intervals on the electrocardiogram (ECG), while prolonged PR interval may be observed in older FD patients [18]. Atrial and ventricular arrhythmias are also relatively common and may be due to atrial myopathy, atrial dilatation from longstanding diastolic dysfunction, and atrial and ventricular fibrosis. Atrial arrhythmias such as atrial fibrillation are more common than ventricular arrhythmias.

In addition, valvular diseases such as aortic, mitral, and tricuspid regurgitation are common in patients with FD due to mild thickening of the valves, although valvular regurgitation significant enough to require intervention is uncommon and stenotic lesions attributed to Fabry disease alone are rare [19]. Thickening of papillary muscles in FD patients has been proposed as a mechanism of mitral regurgitation in FD. Fabry disease can also lead to aortic dilatation, especially in males, where its prevalence increases with age. Aortic dilatation in FD has been shown to be independent of elevated blood pressure [20] and has been attributed to degenerative changes in the aortic media due to excessive glycolipid substrate deposition [21]. Significant aortic dilatation due to Fabry disease resulting in acute aortic events has yet to be reported.

Fabry cardiomyopathy may not be as rare as we once thought as it has been shown to be responsible for up to 4% of unexplained hypertrophic cardiomyopathy (HCM) cases [22,23] and up to 12% of unexplained increased LV wall thickness in other selected cohorts [9]. When assessing for pathogenic mutations only, the prevalence of *GLA* mutation in LVH or HCM clinics is 0.94% in males and 0.90% in females [24]. In fact, the cardiac variant is the most common form of FD in some countries such as Taiwan [25]. This is of particular concern as cardiovascular complications represent the predominant source of FD-related mortality and morbidity [8,26].

## 4. Screening and Diagnosis

A diagnosis of FD is made by demonstrating reduced or absent α-Gal A activity in hemizygous males. In females, genotyping is required as random inactivation of X-chromosome results in mosaicism, resulting in partial expression of the mutated allele that allows for normal levels of α-Gal A activity but still results in Gb_3_ build-up [27].

The prevalence of FD in males was previously estimated to be 1 in 117,000 [28]. However, various newborn screening initiatives around the world such as in Taiwan and Italy have demonstrated a much higher prevalence of disease-causing variants, ranging from 1:1250 to 1:4600, suggesting that FD may be underdiagnosed elsewhere [2,3].

The screening and diagnosis of FD have been simplified with the use of dried blood spot (DBS) testing. Dried blood testing identifies reduced enzyme activity using artificial fluorescent tag substrates linked to an analog of the natural substrate [7]. If enzyme activity is found to be low in male patients, a confirmatory genetic analysis is sent. For female patients, enzyme activity is not a reliable measure of disease activity and therefore all DBS samples are sent for genetic analysis.

## 5. Diagnosis of Fabry Cardiomyopathy

Awareness of the cardiac manifestations of FD may lead to earlier recognition of the condition and differentiation from other causes of increased LV wall thickness. Sensitive cardiac biomarkers and advanced cardiac imaging modalities such as echocardiography with strain imaging and MRI with T1 mapping are essential for the diagnosis and staging of FD.

### 5.1. Echocardiography

**Figure 1:**
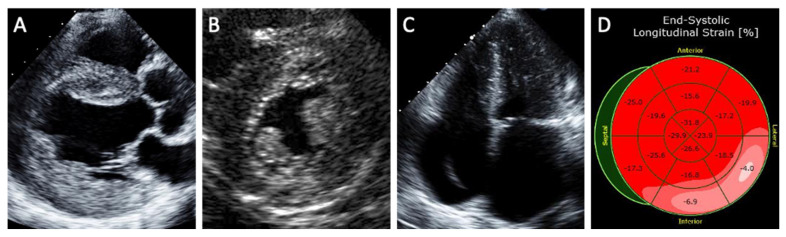
Structural abnormalities detected on echocardiography in patients with Fabry cardiomyopathy. (**A**) Parasternal long-axis view shows increased left ventricular wall thickness (the hallmark feature of Fabry cardiomyopathy) along with thickening of the aortic and mitral valves as well as aortic dilatation. (**B**) Parasternal short-axis view shows prominent papillary muscle and binary sign (hyperechogenic endocardial layer of glycolipid-enriched smooth muscle cells adjacent to a hypoechogenic subendocardial layer relatively devoid of glycolipids). (**C**) Severe biatrial enlargement with a device lead seen in the right-sided chambers for treatment of advanced conduction disease. (**D**) Reduced longitudinal strain in the basal inferolateral segment characteristic of Fabry cardiomyopathy.

Echocardiography is an effective noninvasive method of assessing the degree of cardiac involvement in FD (Figure 1). A concentric pattern of increased LV wall thickness is the hallmark finding of Fabry cardiomyopathy, although other morphologies such as an asymmetric thickening of the interventricular septum, eccentric hypertrophy, and apical hypertrophy have also been described [4]. Other echocardiographic features of Fabry cardiomyopathy include prominent papillary muscles, increased right ventricular wall thickness, atrial enlargement, and the ‘binary sign’. The ‘binary sign’ is a finding characterized by a hyperechogenic endocardial surface composed of glycolipid-enriched smooth muscle cells adjacent to a hypoechogenic subendocardial layer relatively devoid of glycolipids, although recent studies have shown poor sensitivity and specificity of the sign to detect FD [29].

Strain imaging is a sensitive method in identifying subclinical cardiomyopathy. Patients with Fabry cardiomyopathy demonstrate lower global longitudinal strain and circumferential strain compared to healthy subjects [30]. Reduced longitudinal strain in the basal inferolateral segment as well as loss of the base-to-apex circumferential strain gradient have been suggested as specific LV deformation patterns of Fabry cardiomyopathy compared to hypertrophic cardiomyopathy [31].

The application of artificial intelligence (AI) to the echo assessment of patients with increased LV wall thickness may one day facilitate the diagnosis of FD given the known challenges of accurate LV wall thickness measurement [32]. Artificial intelligence-based myocardial texture analysis was suggested as a means of differentiating hypertrophic cardiomyopathy from hypertensive heart disease and uremic cardiomyopathy [33]. Artificial intelligence models have also previously been shown to augment the detection of cardiac amyloidosis and assist in the diagnosis and risk stratification of patients with hypertrophic cardiomyopathy [34,35,36]. The role of AI in assessment of patients with increased wall thickness including patients with possible FD is ongoing in our echocardiography laboratory.

### 5.2. Magnetic Resonance Imaging

Several MRI findings have been described in Fabry cardiomyopathy. Late gadolinium enhancement (LGE) in the basal inferolateral segment is a common MRI finding in Fabry cardiomyopathy and is observed in 50% of affected patients [5]. Shortened myocardial T1 relaxation time can discriminate Fabry cardiomyopathy from other causes of LVH and may be seen in Fabry cardiomyopathy prior to the development of LVH [37,38]. Chronic local T2 elevation in the basal inferolateral segment may indicate myocardial inflammation from Fabry cardiomyopathy and is associated with worse Fabry stabilization index (FASTEX) score [39]. Cardiac MRI can also be helpful in identifying increased right ventricular wall thickness, atrial enlargement, and prominent papillary muscles.

### 5.3. Laboratory Tests

Various laboratory biomarkers have been proposed for use in staging patients with Fabry cardiomyopathy. Troponin level has been correlated with the degree of fibrosis measured by LGE on MRI in patients with Fabry Cardiomyopathy [40]. Increased symptom and disease burden is correlated with elevated levels of CRP, NT-proBNP, and IL-6 [41,42,43].

### 5.4. Cardiopulmonary Exercise Test

Patients with FD have been shown to have decreased heart rate, indexed oxygen pulse, blood pressure, and max VO2 at peak exercise during cardiopulmonary exercise testing using treadmill test and cycle ergometer [44,45,46]. There may be a small improvement in exercise tolerance in patients receiving ERT [44,47].

## 6. Fabry Disease Severity Scores

Several validated scoring systems for FD is available. Fabry disease severity scoring system (DS3) is a validated scoring system utilizing 4 clinical domains: peripheral nervous system, renal, cardiac, and a patient-reported domain. Fabry DS3 has been demonstrated to correlate very well with overall clinical picture of patients with FD using clinical global impression of severity score by FD experts [48]. The Mainz Severity Score Index (MSSI) is another scoring system used to grade severity of disease in FD [49]. The MSSI is composed of four sections (general, neurological, cardiac, and renal) related to symptoms of FD. After one year of treatment with ERT in patients with FD, MSSI was significantly reduced in all patients [49].

The Fabry Stabilization index (FASTEX) is a scoring system developed to assess for clinical stability in patients with FD. The FASTEX was created using consensus weighted score in 28 patients with FD, where the score is based on three domains (nervous system, renal, and cardiac). A worsening global score of ≥20% was suggested to indicate that the patient is clinically unstable [50].

## 7. Treatments in Fabry Disease

The current approach to the treatment and management of FD aims to either prevent or delay the progression of FD to irreversible tissue damage and organ failure. There is currently no curative treatment for FD. To date, treatments available for FD include disease-modifying therapies used in conjunction with non-specific therapies that treat symptoms caused by multi-organ injury. The advantages and disadvantages of currently available as well as investigational FD therapies are summarized in Table 3.

Enzyme replacement therapy (ERT) is currently the standard treatment for males with classical FD and Type 2 non-classical FD, and females with classical FD. Enzyme replacement therapy became available in 2001 and represents the first treatment developed for FD. Two formulations of ERT currently exist: agalsidase α (Replagal) administered at a dose of 0.2 mg/kg intravenously every two weeks and agalsidase β (Fabrazyme) administered at a dose of 1 mg/kg intravenously every two weeks. Agalsidase α is generated from a continuous human cell line with the activation of the *GLA* gene, while agalsidase β is generated from a Chinese hamster ovary mammalian cell expression system transduced with the human *GLA* sequence. Enzyme replacement therapy has been shown to effectively reduce glycolipid substrates including Gb_3_ in the urine, plasma, and tissues of patients with FD [51]. With respect to FD-related cardiac injury, ERT has been shown to effectively reduce Gb_3_ inclusions in endothelial cells, with less clear evidence regarding Gb_3_ clearance from cardiomyocytes [52]. In addition, observational studies have reported a reduction in LV wall thickness in patients treated with ERT [53,54].

The limitations of ERT include the short plasma half-life of the recombinant enzyme, thus necessitating bi-weekly infusions and that it can only delay the progression of FD. Enzyme replacement therapy also has limited efficacy in later stages of Fabry cardiomyopathy, when fibrosis is already present [53], and it is unclear whether or not ERT slows the progression of fibrosis [55]. Additionally, anti-drug antibodies against the recombinant replacement enzyme in ERT has been reported to develop in 64–88% of FD patients [52,56], thereby attenuating the effect of ERT. Finally, ERT demonstrates uneven biodistribution, with the liver taking up the majority of the recombinant replacement enzyme, whereas the most severely affected cell types in the body such as cardiomyocytes and podocytes take up lesser amounts of the replacement enzyme [55].

There is currently no evidence demonstrating the superiority of agalsidase α over agalsidase β and vice versa in clinical endpoints [57,58]. Specifically, in the Canadian Fabry Disease Initiative, a comparison of agalsidase α and agalsidase β demonstrated no statistical difference in clinical endpoints including death, cardiac events, acute neurological events, and others [58]. However, there were differences in the biochemical response between patients treated with agalsidase α and agalsidase β, with a higher risk of developing anti-drug antibodies and a greater decrease in the plasma globotriaosylsphingosine levels in patients treated with agalsidase β. In addition, there was a greater reduction in the left ventricular mass in patients treated with agalsidase β [59].

Oral pharmacologic chaperone therapy, namely Migalastat, is an alternative treatment option for FD. However, since Migalastat is protein-variant specific, it is only used for patients with amenable *GLA* gene variants [60]. These specific *GLA* variants produce highly unstable mutated α-Gal A proteins. Migalastat binds to these α-Gal A variants, thus stabilizing the enzymes by enhancing correct folding [55]. This stabilization allows the mutated enzymes to be properly trafficked to lysosomes, where Migalastat dissociates, allowing it to catabolize the accumulated Gb_3_ substrates [61]. This therapy has been demonstrated to both increase α-Gal A activity and decrease Gb_3_ inclusions [62,63].

Despite disease-modifying FD treatments described, equal attention and care should be given to non-FD specific treatments directed towards the multi-system consequences of the condition. Due to the clinical heterogeneity of FD, a multidisciplinary clinical team with a cardiologist, nephrologist, neurologist, genetic counselor, and a medical geneticist should ideally be in place for the holistic care of FD patients. General preventative measures including pharmacological stroke prophylaxis with an antithrombic agent and lifestyle modifications such as avoidance of extremes of temperature to prevent painful crises, exercise prescription, diet, and smoking cessation should be appropriately advised. Other co-morbidities such as hypertension and dyslipidemia should be managed diligently. The management of the cardiac manifestations of FD has been summarized in Table 4.

## 8. Future Directions in the Management of Fabry Disease

There are three potential future treatment options currently undergoing preclinical investigation: (1) second-generation ERT; (2) substrate reduction therapy (SRT); and (3) gene therapy.

To date, two formulations of second-generation ERTs have been developed: pegunigalsidase-α and moss-aGal. These second-generation ERTs are plant-derived with different pharmacokinetic properties that may lead to better biodistribution in the body compared with first-generation ERTs.

Substrate reduction therapy including Venglustat and Lucerastat are potential future oral treatments for FD. Substrate reduction therapy limits the formation of pathogenic metabolites such as Gb_3,_ thus limiting its accumulation in tissues throughout the body [64]. Substrate reduction therapy may have a potential role as an adjunctive therapy used alongside ERT [64].

Gene therapy may be a treatment option that enables FD patients to receive fewer treatments with more permanent effects. This novel therapy involves the encapsulation of mRNA into lipid nanoparticles that target hepatocytes where endogenous protein translation, glycosylation, and intracellular trafficking of α-Gal A occurs. Functional α-Gal A enzymes are then subsequently secreted into the circulation, which can be taken up by affected cells [65]. Recently, the first gene therapy trial for FD was conducted, where classical FD patients were infused with lentivirus-transduced hematopoietic stem cells engineered to express α-Gal A [51]. All patients produced α-Gal A to near normal levels within one week of therapy with observations of reduced plasma and urine Gb_3_ [51].

Several challenges exist in the early identification of individuals with FD. Many non-classic FD patients are asymptomatic in their early years of life, resulting in delayed diagnosis. Additionally, remote centers may not have the capacity for advanced imaging. Even when presented with imaging findings of FD, clinicians may not readily diagnose FD due to the non-specific findings and unfamiliarity with the clinical manifestations of FD.

To overcome these challenges, our echocardiography lab has undertaken several initiatives that aim to achieve earlier detection of Fabry cardiomyopathy, including the development of artificial intelligence (AI)-empowered detection of increased LV wall thickness, the use of DBS testing at the time of echocardiography when an unexplained increase in LV wall thickness is identified, and the dissemination of point-of-care ultrasound devices to community healthcare providers to promote widespread screening for the presence of cardiomyopathies [66,67,68].

The use of AI to effectively identify increased LV wall thickness and differentiate between the many possible etiologies is a promising area for further research. An AI approach based on 12-lead ECG has been shown to be 87% sensitive and 90% specific in identifying hypertrophic cardiomyopathy [69]. Fully automated echocardiographic interpretation has also been shown to be capable of detecting hypertrophic cardiomyopathy and cardiac amyloidosis with C-statistics of 0.93 and 0.87 respectively [36]. An AI strategy based on late gadolinium enhancement (LGE) patterns on cardiac MRI demonstrated 88% diagnostic accuracy in detecting cardiac amyloidosis [70]. Further research is needed to determine the potential role of AI in the diagnosis of Fabry disease.

## 9. Conclusions and Call to Action

Our understanding of the pathophysiology, diagnosis and treatment of FD continue to rapidly evolve. We now have not only the capability to more effectively diagnose FD using various laboratory and imaging modalities, but also effective treatment options for the condition. Clinicians should have an understanding of the clinical manifestations of FD and consider it as part of the differential diagnosis when presented with unexplained increased LV wall thickness. Fabry disease should no longer be considered a rare, untreatable disease, but one that can and should be identified and treated in a timely manner.

## Figures and Tables

**Table 1:** Cardiac manifestations of Fabry disease.

Structural abnormalities detected by cardiac imaging
Increased LV wall thickness. Morphologies include concentric hypertrophy (most common), asymmetric septal hypertrophy, eccentric hypertrophy, and apical hypertrophy. Associated LV outflow tract obstruction may be present but often not [4] LV ejection fraction often preserved but may be reduced with advanced disease [4] Biatrial enlargement due to chronic diastolic dysfunction or underlying atrial myopathy [4] Prominent papillary muscles [4] Reduced LV longitudinal strain on echocardiography and T2 elevation (suggesting inflammation) or late gadolinium enhancement (suggesting fibrosis) on MRI in the basal inferolateral segment [4] Reduced native T1 values on MRI [4] Abnormal LV diastolic function [4] Binary sign (no longer considered sensitive or specific for Fabry cardiomyopathy) [4] RV wall thickness may be increased [4] Thickening and redundancy of the valves with some degree of valvular regurgitation, although often not significant enough to warrant intervention [4] Aortic dilatation [4]
Electrophysiologic abnormalities detected by ECG or prolonged rhythm monitoring
Short PR interval in younger patients, prolonged PR interval in older patients [14] Bradycardia from chronotropic incompetence [15] Sinus node dysfunction [15] Varying degrees of AV block [15] Atrial arrhythmias including atrial tachycardia, atrial flutter, or atrial fibrillation [15] Ventricular arrhythmias including non-sustained VT and sustained VT [15]

Abbreviations: AV, atrioventricular; ECG, electrocardiogram; LV, left ventricular; MRI, magnetic resonance imaging; RV, right ventricular; VT, ventricular tachycardia.

**Table 2:** Differential diagnosis of increased LV wall thickness and common findings on patient history, ECG, echocardiography, and CMR.

	Patient History	ECG	Echocardiography	CMR
**Fabry Cardiomyopathy**	Angiokeratoma corporis Acroparesthesia Diarrhea Stroke Chest pain Heart failure	Short PR interval Prolonged QRS High voltage QRS	Prominent PMBinary sign Loss of base-to-apex circumferential strain gradient	LGE and T2 increase in basal inferolateral wall Shortened T1 relaxation time
**Hypertension**	History of hypertension	High voltage QRS	Concentric LVH Diastolic dysfunction Reduced LV GLS	Absence of LGE
**Athlete’s Heart**	Asymptomatic History of sporting activity Resting bradycardia	Normal Possible high voltage QRS	Ventricular hypertrophy and dilatation	Absence of LGE Normal LV SVI
**Aortic Stenosis**	Chest pain Dyspnea Syncope	High voltage QRS Left atrial enlargement	Aortic stenosis Concentric LVH	Focal mid-wall LGE
**Hypertrophic Cardiomyopathy**	Chest pain Dyspnea	High voltage QRS Left atrial enlargement Atrial fibrillation	Asymmetric septal hypertrophy LVOT obstruction Systolic anterior motion	Patchy mid-wall LGE
**Cardiac Amyloidosis**	Heart failure Bilateral carpal tunnel Nephrotic syndrome Macroglossia Peripheral neuropathy	Low voltage QRS Atrial fibrillation Pseudo-infarct	Bi-atrial enlargement Diastolic dysfunction Abnormal LV GLS in mid and basal walls with apical sparing	Global subendocardial LGE Abnormal myocardial and blood-pool gadolinium kinetics

Abbreviations: CMR, cardiovascular magnetic resonance imaging; ECG, electrocardiogram; GLS, global longitudinal strain; LGE, late gadolinium enhancement; LV, left ventricular; LVH, left ventricular hypertrophy, LVOT, left ventricular outflow tract; PM, papillary muscle; SVI, stroke volume index.

**Table 3:** Comparison of approved and investigational disease-modifying therapies for Fabry disease.

Disease-Modifying Therapy	Advantages	Disadvantages
First-generation ERT Agalsidase α (Replagal) Agalsidase β (Fabrazyme)	Nearly two decades of clinical experience Applicable to most patients with FD Effectively reduces Gb3 in urine, plasma, tissues, and endothelial cells Reduction in LV wall thickness seen in observational studies	Lifelong biweekly intravenous infusions Anti-drug antibodies may developExpensive Less clear evidence regarding Gb3 clearance from cardiomyocytes Uneven biodistribution resulting in limited uptake in cardiomyocytes and podocytes Unclear effects on fibrosis possibly limiting its efficacy in later stages of FD Non-curative
Oral chaperone therapy Migalastat	Oral route of administration Increases α-Gal A activity Decrease Gb3 inclusions	Only applicable to certain GLA variants Approval limited to certain countries Variable therapeutic response Non-curative
Second-generation ERT Pegunigalsidase-α Moss-aGal	Possible monthly (vs. biweekly) schedule Improved biodistribution compared with first-generation ERT	Intravenous route of administration Currently in preclinical phase Non-curative
Substrate reduction therapy Venglustat Lucerastat	Oral route of administration Can be used as adjunctive therapy to ERT	Cannot be used as a monotherapy Currently in preclinical phase Non-curative
Gene therapy	Longer lasting therapeutic effects Potentially curative although still uncertain	Uncertain long-term adverse effects Currently in pre-clinical phase

**Table 4:** Management of the cardiovascular manifestations of Fabry disease.

Structural abnormalities that can be present on cardiac imaging
Identification/treatment of hypertension to prevent further increase in LV wall thickness Cautious use of medications with negative inotropic effects (e.g., beta-blockers, non-dihydropyridine calcium channel blockers, disopyramide) if LVOT obstruction present Regular surveillance of valvular regurgitation and aortic dilatation
Electrophysiologic abnormalities detected by ECG or rhythm monitoring
Atrial arrhythmias may require treatment with rate control strategy (using AV nodal blockers) or rhythm control strategy (with anti-arrhythmic agents or catheter ablation) Anticoagulation for stroke risk reduction is indicated in the setting of atrial fibrillation Ventricular arrhythmias may require treatment with either beta-blockers, anti-arrhythmic agents, catheter ablation, or ICD therapy Amiodarone should only be considered when other therapeutic options have failed since it may promote glycolipid accumulation and attenuate the effects of ERT Symptomatic bradyarrhythmias including sinus node dysfunction or advanced AV block may require permanent pacing
Other cardiovascular considerations in patients with Fabry disease
ACE inhibitors or ARBs should be considered for patients with chronic kidney disease Beta-blockers or non-dihydropyridine calcium-channel blockers should be used with caution given the higher prevalence of sinus node dysfunction or advanced AV block Antiplatelet therapy may be indicated for primary or secondary prevention of ischemic stroke in selected patients Heart failure can be treated with diuretics or LV enhancement therapy if LVEF is reduced in accordance with contemporary consensus-based guidelines Chest pain due to large-vessel or microvascular disease can be managed with standard anti-anginal therapy with cautious use of AV nodal blockers as previously described

Abbreviations: ACE, angiotensin converting enzyme; ARB, angiotensin II receptor blocker; AV, atrioventricular; ERT, enzyme replacement therapy; ICD, implantable cardioverter-defibrillator; LV, left ventricular; LVEF, left ventricular ejection fraction; LVOT, left ventricular outflow tract.

## Abbreviations

a-Gal A	α-galactosidase A
ACE	Angiotensin converting enzyme
ARB	Angiotensin II receptor blocker
AI	Artificial intelligence
AV	Atrioventricular
DBS	Dried blood spot
ECG	Electrocardiogram
ERT	Enzyme replacement therapy
FD	Fabry disease
Gb_3_	Globotriaosylceramide
HCM	Hypertrophic cardiomyopathy
ICD	Implantable cardioverter-defibrillator
LGE	Late gadolinium enhancement
LV	Left ventricular
LVEF	Left ventricular ejection fraction
LVOT	Left ventricular outflow tract
MRI	Magnetic resonance imaging
RV	Right ventricular
SRT	Substrate reduction therapy
VT	Ventricular tachycardia
